# 3-(4-Amino-3-ethyl-5-sulfanyl­idene-4,5-dihydro-1*H*-1,2,4-triazol-1-yl)-3-(2-chloro­phen­yl)-1-phenyl­propan-1-one

**DOI:** 10.1107/S1600536811025803

**Published:** 2011-07-06

**Authors:** Zhi-jian Wang, Wei-min Jia, Qing-lei Liu, Wei Wang

**Affiliations:** a School of Perfume and Aroma Technology, Shanghai Istitute of Technology, Shanghai 200235, People’s Republic of China; b School of Chemical Engineering, University of Science and Technology LiaoNing, Anshan 114051, People’s Republic of China

## Abstract

In the title mol­ecule, C_19_H_19_ClN_4_OS, the 1,2,4-triazole ring forms dihedral angles of 86.0 (2) and 65.6 (2)° with the phenyl and chloro­phenyl rings, respectively. In the crystal, inter­molecular N—H⋯S and N—H⋯O hydrogen bonds link mol­ecules into centrosymmetric dimers, which are further linked into chains in [001] *via* weak C—H⋯π inter­actions.

## Related literature

For the crystal structures of related 1,2,4-triazole-5(4*H*)-thione derivates, see: Al-Tamimi *et al.* (2010 ▶); Fun *et al.* (2009 ▶); Gao *et al.* (2011 ▶); Tan *et al.* (2010 ▶); Wang *et al.* (2011 ▶).
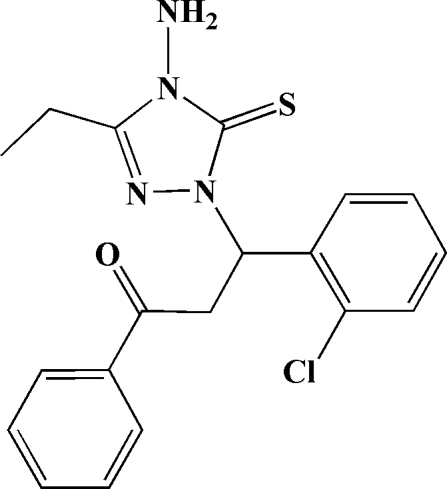


## Experimental

### 

#### Crystal data

C_19_H_19_ClN_4_OS
*M* *_r_* = 386.89
Monoclinic, 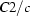

*a* = 29.023 (10) Å
*b* = 7.561 (2) Å
*c* = 18.286 (6) Å
β = 109.606 (6)°
*V* = 3780 (2) Å^3^
*Z* = 8
Mo *K*α radiation
μ = 0.33 mm^−1^
*T* = 113 K
0.20 × 0.18 × 0.12 mm

#### Data collection

Rigaku Saturn CCD area-detector diffractometer
Absorption correction: multi-scan (*CrystalClear*; Rigaku/MSC, 2005 ▶) *T*_min_ = 0.937, *T*_max_ = 0.962
22962 measured reflections
4441 independent reflections
3430 reflections with *I* > 2σ(*I*)
*R*_int_ = 0.036

#### Refinement

*R*[*F* ^2^ > 2σ(*F* ^2^)] = 0.033
*wR*(*F* ^2^) = 0.088
*S* = 1.02
4441 reflections
244 parameters
H atoms treated by a mixture of independent and constrained refinement
Δρ_max_ = 0.37 e Å^−3^
Δρ_min_ = −0.23 e Å^−3^

### 

Data collection: *CrystalClear* (Rigaku/MSC, 2005 ▶); cell refinement: *CrystalClear*; data reduction: *CrystalClear*; program(s) used to solve structure: *SHELXS97* (Sheldrick, 2008 ▶); program(s) used to refine structure: *SHELXL97* (Sheldrick, 2008 ▶); molecular graphics: *SHELXTL* (Sheldrick, 2008 ▶); software used to prepare material for publication: *SHELXTL*.

## Supplementary Material

Crystal structure: contains datablock(s) global, I. DOI: 10.1107/S1600536811025803/cv5121sup1.cif

Structure factors: contains datablock(s) I. DOI: 10.1107/S1600536811025803/cv5121Isup2.hkl

Supplementary material file. DOI: 10.1107/S1600536811025803/cv5121Isup3.cml

Additional supplementary materials:  crystallographic information; 3D view; checkCIF report

## Figures and Tables

**Table 1:** Hydrogen-bond geometry (Å, °) *Cg* is the centroid of the C12–C17 ring.

*D*—H⋯*A*	*D*—H	H⋯*A*	*D*⋯*A*	*D*—H⋯*A*
N4—H4*B*⋯S1	0.892 (18)	2.763 (16)	3.2239 (16)	113.5 (12)
N4—H4*A*⋯S1^i^	0.914 (18)	2.608 (18)	3.4773 (16)	159.2 (14)
N4—H4*A*⋯O1^i^	0.914 (18)	2.602 (18)	2.9123 (16)	100.6 (14)
N4—H4*B*⋯O1^i^	0.892 (18)	2.466 (15)	2.9123 (16)	111.3 (12)
C10—H10⋯*Cg*^ii^	0.95	2.62	3.508 (2)	156

Symmetry codes: (i) 
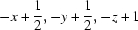
; (ii) 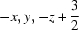.

**Fig. 1.**
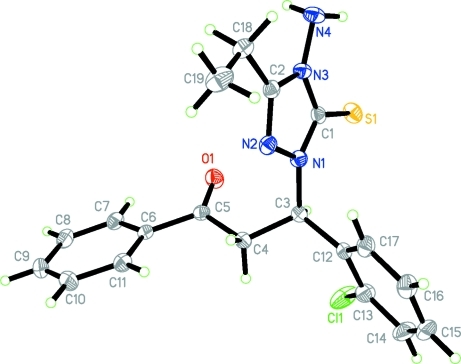
View of the molecule of (I) showing the atom-labelling scheme. Displacement ellipsoids are drawn at the 55% probability level.

## supplementary crystallographic information

### Comment

In continuation of structural study of 1,2,4-triazole-5(4*H*)-thione derivatives in our group (Wang *et al.*, 2011; Gao *et al.*, 2011), we present here the crystal structure of the title compound, (I).

The bond lengths and angles in compound (I) are found to have normal values comparable with those reported in the related 1,2,4-triazole-5(4*H*)- thione derivatives (Al-Tamimi *et al.*, 2010; Fun *et al.* (2009); Tan *et al.* (2010); Wang *et al.*, 2011;  Gao *et al.*, 2011). The 1,2,4-triazole ring makes the dihedral angles of 94.0 (2) and 65.6 (2)Å with the phenyl ring and the chlorophenyl ring, respectively. The phenyl ring and the chlorophenyl ring form a dihedral angle of 81.8 (2) Å.

In the crystal structure, intermolecular N—H···S and N—H···O (Table 1) hydrogen bonds link molecules into centrosymmetric dimers, which are further linked into chains in [001] *via* the weak C—H···π interactions (Table 1).

### Experimental

The title compound was synthesized by the reaction of the 3-(2-chlorophenyl) -1-phenyl-2-propen-1-one (2.0 mmol) with 4-amino-3-ethyl-4*H*-1,2,4-triazole- 5-thiol (2.0 mmol) in ethanol. The reaction progress was monitored *via* TLC. The resulting precipitate was filtered off, washed with cold ethanol, dried and purified to give the target product as colorless solid in 74% yield. Crystals of (I) suitable for single-crystal X-ray analysis were grown by slow evaporation of a solution in chloroform-ethanol (1:1).

### Refinement

The H atoms attached to N atoms were located in a different density map and the atomic coordinations allowed to refine freely. Other H atoms were positioned geometrically and refined as riding (C—H = 0.95–1.00 Å) and allowed to ride on their parent atoms, with *U*_iso_(H) = 1.2-1.5 *U*_eq_ of the parent atom.

### Crystal data

**Table:**

C_19_H_19_ClN_4_OS	*F*(000) = 1616
*M**_r_* = 386.89	*D*_x_ = 1.360 Mg m^−^^3^
Monoclinic, *C*2/*c*	Mo *K*α radiation, λ = 0.71073 Å
Hall symbol: -C 2yc	Cell parameters from 6659 reflections
*a* = 29.023 (10) Å	θ = 1.5–27.9°
*b* = 7.561 (2) Å	µ = 0.33 mm^−^^1^
*c* = 18.286 (6) Å	*T* = 113 K
β = 109.606 (6)°	Prism, colourless
*V* = 3780 (2) Å^3^	0.20 × 0.18 × 0.12 mm
*Z* = 8	

### Data collection

**Table:**

Rigaku Saturn CCD area-detector diffractometer	4441 independent reflections
Radiation source: rotating anode	3430 reflections with *I* > 2σ(*I*)
multilayer	*R*_int_ = 0.036
Detector resolution: 14.22 pixels mm^-1^	θ_max_ = 27.8°, θ_min_ = 1.5°
φ and ω scans	*h* = −38→38
Absorption correction: multi-scan (*CrystalClear*; Rigaku/MSC, 2005)	*k* = −9→9
*T*_min_ = 0.937, *T*_max_ = 0.962	*l* = −23→23
22962 measured reflections	

### Refinement

**Table:**

Refinement on *F*^2^	Primary atom site location: structure-invariant direct methods
Least-squares matrix: full	Secondary atom site location: difference Fourier map
*R*[*F*^2^ > 2σ(*F*^2^)] = 0.033	Hydrogen site location: inferred from neighbouring sites
*wR*(*F*^2^) = 0.088	H atoms treated by a mixture of independent and constrained refinement
*S* = 1.02	*w* = 1/[σ^2^(*F*_o_^2^) + (0.0519*P*)^2^] where *P* = (*F*_o_^2^ + 2*F*_c_^2^)/3
4441 reflections	(Δ/σ)_max_ = 0.001
244 parameters	Δρ_max_ = 0.37 e Å^−^^3^
0 restraints	Δρ_min_ = −0.23 e Å^−^^3^

### Special details

**Table:**

Geometry. All e.s.d.'s (except the e.s.d. in the dihedral angle between two l.s. planes) are estimated using the full covariance matrix. The cell e.s.d.'s are taken into account individually in the estimation of e.s.d.'s in distances, angles and torsion angles; correlations between e.s.d.'s in cell parameters are only used when they are defined by crystal symmetry. An approximate (isotropic) treatment of cell e.s.d.'s is used for estimating e.s.d.'s involving l.s. planes.
Refinement. Refinement of *F*^2^ against ALL reflections. The weighted *R*-factor *wR* and goodness of fit *S* are based on *F*^2^, conventional *R*-factors *R* are based on *F*, with *F* set to zero for negative *F*^2^. The threshold expression of *F*^2^ > σ(*F*^2^) is used only for calculating *R*-factors(gt) *etc*. and is not relevant to the choice of reflections for refinement. *R*-factors based on *F*^2^ are statistically about twice as large as those based on *F*, and *R*- factors based on ALL data will be even larger.

### Fractional atomic coordinates and isotropic or equivalent isotropic displacement parameters (Å^2^)

**Table:**

	*x*	*y*	*z*	*U*_iso_*/*U*_eq_	
S1	0.190030 (13)	0.38390 (4)	0.542685 (19)	0.02176 (10)	
Cl1	0.019191 (13)	0.34916 (5)	0.39368 (3)	0.03607 (12)	
O1	0.15205 (3)	0.13842 (11)	0.34074 (5)	0.0218 (2)	
N1	0.16509 (4)	0.51514 (13)	0.39437 (6)	0.0155 (2)	
N2	0.18602 (4)	0.59175 (14)	0.34375 (6)	0.0168 (2)	
N3	0.24120 (4)	0.53762 (13)	0.45809 (6)	0.0157 (2)	
N4	0.28746 (4)	0.53417 (16)	0.51708 (7)	0.0214 (3)	
C1	0.19819 (5)	0.47776 (16)	0.46471 (7)	0.0155 (3)	
C2	0.23249 (5)	0.60456 (15)	0.38496 (7)	0.0159 (3)	
C3	0.11395 (4)	0.45895 (16)	0.36740 (7)	0.0158 (3)	
H3	0.1105	0.3619	0.4024	0.019*	
C4	0.10039 (5)	0.38305 (16)	0.28491 (7)	0.0175 (3)	
H4C	0.0647	0.3617	0.2643	0.021*	
H4D	0.1083	0.4716	0.2510	0.021*	
C5	0.12692 (4)	0.21139 (16)	0.28159 (7)	0.0159 (3)	
C6	0.11887 (4)	0.13029 (16)	0.20350 (7)	0.0159 (3)	
C7	0.12856 (4)	−0.05123 (17)	0.20020 (8)	0.0188 (3)	
H7	0.1405	−0.1181	0.2468	0.023*	
C8	0.12069 (5)	−0.13266 (17)	0.12925 (8)	0.0217 (3)	
H8	0.1269	−0.2557	0.1273	0.026*	
C9	0.10383 (5)	−0.03574 (19)	0.06100 (8)	0.0255 (3)	
H9	0.0985	−0.0924	0.0125	0.031*	
C10	0.09476 (5)	0.14466 (19)	0.06362 (8)	0.0255 (3)	
H10	0.0837	0.2115	0.0170	0.031*	
C11	0.10196 (5)	0.22674 (18)	0.13465 (8)	0.0206 (3)	
H11	0.0953	0.3494	0.1363	0.025*	
C12	0.07870 (5)	0.60609 (16)	0.36975 (7)	0.0163 (3)	
C13	0.03355 (5)	0.56770 (17)	0.37776 (8)	0.0216 (3)	
C14	−0.00095 (5)	0.69742 (19)	0.37456 (9)	0.0270 (3)	
H14	−0.0313	0.6667	0.3801	0.032*	
C15	0.00937 (5)	0.87208 (18)	0.36319 (8)	0.0249 (3)	
H15	−0.0141	0.9619	0.3602	0.030*	
C16	0.05417 (5)	0.91481 (18)	0.35614 (8)	0.0227 (3)	
H16	0.0614	1.0345	0.3485	0.027*	
C17	0.08856 (5)	0.78348 (17)	0.36023 (7)	0.0196 (3)	
H17	0.1194	0.8153	0.3565	0.024*	
C18	0.27199 (5)	0.67570 (18)	0.35804 (8)	0.0203 (3)	
H18A	0.2920	0.5760	0.3503	0.024*	
H18B	0.2936	0.7530	0.3990	0.024*	
C19	0.25249 (5)	0.7808 (2)	0.28262 (9)	0.0284 (3)	
H19A	0.2305	0.7060	0.2420	0.043*	
H19B	0.2799	0.8195	0.2666	0.043*	
H19C	0.2345	0.8845	0.2908	0.043*	
H4A	0.3003 (6)	0.425 (2)	0.5136 (10)	0.040 (5)*	
H4B	0.2814 (6)	0.522 (2)	0.5616 (10)	0.035 (5)*	

### Atomic displacement parameters (Å^2^)

**Table:**

	*U*^11^	*U*^22^	*U*^33^	*U*^12^	*U*^13^	*U*^23^
S1	0.02654 (19)	0.02354 (19)	0.01549 (17)	−0.00294 (14)	0.00745 (13)	0.00238 (13)
Cl1	0.02154 (19)	0.01854 (19)	0.0719 (3)	−0.00307 (14)	0.02069 (19)	−0.00139 (18)
O1	0.0255 (5)	0.0209 (5)	0.0170 (5)	0.0044 (4)	0.0045 (4)	0.0016 (4)
N1	0.0157 (5)	0.0170 (5)	0.0144 (5)	0.0003 (4)	0.0059 (4)	0.0017 (4)
N2	0.0180 (5)	0.0179 (6)	0.0162 (5)	0.0003 (4)	0.0081 (4)	0.0016 (4)
N3	0.0140 (5)	0.0153 (5)	0.0161 (5)	0.0004 (4)	0.0028 (4)	0.0008 (4)
N4	0.0163 (6)	0.0235 (7)	0.0192 (6)	0.0007 (5)	−0.0010 (5)	0.0016 (5)
C1	0.0179 (6)	0.0130 (6)	0.0152 (6)	0.0001 (5)	0.0049 (5)	−0.0023 (5)
C2	0.0174 (6)	0.0132 (6)	0.0170 (6)	0.0026 (5)	0.0057 (5)	−0.0003 (5)
C3	0.0150 (6)	0.0163 (6)	0.0156 (6)	−0.0007 (5)	0.0045 (5)	−0.0018 (5)
C4	0.0169 (6)	0.0188 (7)	0.0152 (6)	0.0018 (5)	0.0034 (5)	−0.0019 (5)
C5	0.0126 (6)	0.0175 (6)	0.0182 (6)	−0.0024 (5)	0.0060 (5)	−0.0015 (5)
C6	0.0116 (6)	0.0195 (7)	0.0170 (6)	−0.0001 (5)	0.0052 (5)	−0.0017 (5)
C7	0.0173 (7)	0.0196 (7)	0.0219 (7)	−0.0008 (5)	0.0097 (5)	0.0001 (5)
C8	0.0200 (7)	0.0199 (7)	0.0285 (7)	−0.0024 (5)	0.0124 (6)	−0.0054 (6)
C9	0.0231 (7)	0.0336 (8)	0.0204 (7)	0.0000 (6)	0.0081 (6)	−0.0092 (6)
C10	0.0251 (7)	0.0329 (8)	0.0162 (6)	0.0063 (6)	0.0039 (6)	0.0012 (6)
C11	0.0199 (7)	0.0205 (7)	0.0210 (7)	0.0044 (5)	0.0065 (5)	−0.0007 (5)
C12	0.0168 (6)	0.0183 (7)	0.0126 (6)	0.0008 (5)	0.0034 (5)	−0.0026 (5)
C13	0.0182 (7)	0.0168 (7)	0.0282 (7)	−0.0029 (5)	0.0056 (6)	−0.0038 (6)
C14	0.0148 (7)	0.0259 (8)	0.0401 (9)	−0.0008 (6)	0.0087 (6)	−0.0052 (7)
C15	0.0211 (7)	0.0216 (7)	0.0304 (8)	0.0071 (6)	0.0068 (6)	−0.0024 (6)
C16	0.0296 (8)	0.0168 (7)	0.0227 (7)	0.0026 (6)	0.0102 (6)	0.0017 (6)
C17	0.0226 (7)	0.0209 (7)	0.0178 (6)	0.0011 (5)	0.0101 (5)	0.0008 (5)
C18	0.0172 (6)	0.0221 (7)	0.0232 (7)	0.0013 (5)	0.0090 (5)	0.0012 (6)
C19	0.0258 (8)	0.0328 (8)	0.0307 (8)	0.0029 (6)	0.0147 (6)	0.0111 (7)

### Geometric parameters (Å, °)

**Table:**

S1—C1	1.6792 (14)	C8—C9	1.387 (2)
Cl1—C13	1.7521 (14)	C8—H8	0.9500
O1—C5	1.2151 (15)	C9—C10	1.393 (2)
N1—C1	1.3519 (16)	C9—H9	0.9500
N1—N2	1.3922 (14)	C10—C11	1.3906 (19)
N1—C3	1.4613 (16)	C10—H10	0.9500
N2—C2	1.3092 (16)	C11—H11	0.9500
N3—C1	1.3709 (16)	C12—C17	1.3945 (17)
N3—C2	1.3716 (16)	C12—C13	1.3970 (19)
N3—N4	1.4123 (15)	C13—C14	1.3886 (19)
N4—H4A	0.914 (18)	C14—C15	1.385 (2)
N4—H4B	0.892 (18)	C14—H14	0.9500
C2—C18	1.4916 (18)	C15—C16	1.3873 (19)
C3—C12	1.5218 (17)	C15—H15	0.9500
C3—C4	1.5366 (17)	C16—C17	1.3920 (19)
C3—H3	1.0000	C16—H16	0.9500
C4—C5	1.5207 (17)	C17—H17	0.9500
C4—H4C	0.9900	C18—C19	1.5265 (19)
C4—H4D	0.9900	C18—H18A	0.9900
C5—C6	1.4987 (17)	C18—H18B	0.9900
C6—C11	1.3939 (18)	C19—H19A	0.9800
C6—C7	1.4062 (18)	C19—H19B	0.9800
C7—C8	1.3840 (18)	C19—H19C	0.9800
C7—H7	0.9500		
			
C1—N1—N2	112.97 (10)	C8—C9—C10	120.00 (13)
C1—N1—C3	125.26 (11)	C8—C9—H9	120.0
N2—N1—C3	121.12 (10)	C10—C9—H9	120.0
C2—N2—N1	104.06 (10)	C11—C10—C9	119.88 (13)
C1—N3—C2	109.41 (10)	C11—C10—H10	120.1
C1—N3—N4	125.93 (11)	C9—C10—H10	120.1
C2—N3—N4	124.65 (11)	C10—C11—C6	120.47 (12)
N3—N4—H4A	105.6 (10)	C10—C11—H11	119.8
N3—N4—H4B	105.7 (10)	C6—C11—H11	119.8
H4A—N4—H4B	100.5 (14)	C17—C12—C13	116.87 (11)
N1—C1—N3	102.97 (11)	C17—C12—C3	122.10 (12)
N1—C1—S1	129.69 (10)	C13—C12—C3	120.95 (11)
N3—C1—S1	127.34 (10)	C14—C13—C12	122.49 (13)
N2—C2—N3	110.57 (11)	C14—C13—Cl1	117.70 (11)
N2—C2—C18	126.44 (12)	C12—C13—Cl1	119.81 (10)
N3—C2—C18	122.97 (11)	C15—C14—C13	119.38 (13)
N1—C3—C12	113.22 (10)	C15—C14—H14	120.3
N1—C3—C4	109.66 (10)	C13—C14—H14	120.3
C12—C3—C4	110.33 (10)	C14—C15—C16	119.51 (12)
N1—C3—H3	107.8	C14—C15—H15	120.2
C12—C3—H3	107.8	C16—C15—H15	120.2
C4—C3—H3	107.8	C15—C16—C17	120.43 (13)
C5—C4—C3	113.05 (10)	C15—C16—H16	119.8
C5—C4—H4C	109.0	C17—C16—H16	119.8
C3—C4—H4C	109.0	C16—C17—C12	121.28 (13)
C5—C4—H4D	109.0	C16—C17—H17	119.4
C3—C4—H4D	109.0	C12—C17—H17	119.4
H4C—C4—H4D	107.8	C2—C18—C19	113.14 (11)
O1—C5—C6	121.16 (11)	C2—C18—H18A	108.9
O1—C5—C4	120.85 (11)	C19—C18—H18A	108.9
C6—C5—C4	117.91 (10)	C2—C18—H18B	109.0
C11—C6—C7	119.13 (12)	C19—C18—H18B	108.9
C11—C6—C5	122.79 (11)	H18A—C18—H18B	107.8
C7—C6—C5	118.08 (11)	C18—C19—H19A	109.5
C8—C7—C6	120.12 (12)	C18—C19—H19B	109.5
C8—C7—H7	119.9	H19A—C19—H19B	109.5
C6—C7—H7	119.9	C18—C19—H19C	109.5
C7—C8—C9	120.39 (13)	H19A—C19—H19C	109.5
C7—C8—H8	119.8	H19B—C19—H19C	109.5
C9—C8—H8	119.8		
			
C1—N1—N2—C2	−1.44 (13)	C4—C5—C6—C7	−158.52 (11)
C3—N1—N2—C2	−172.66 (10)	C11—C6—C7—C8	−0.87 (18)
N2—N1—C1—N3	1.50 (13)	C5—C6—C7—C8	178.65 (11)
C3—N1—C1—N3	172.29 (10)	C6—C7—C8—C9	0.85 (19)
N2—N1—C1—S1	−179.21 (9)	C7—C8—C9—C10	0.1 (2)
C3—N1—C1—S1	−8.41 (19)	C8—C9—C10—C11	−0.9 (2)
C2—N3—C1—N1	−0.99 (13)	C9—C10—C11—C6	0.9 (2)
N4—N3—C1—N1	178.19 (11)	C7—C6—C11—C10	−0.01 (19)
C2—N3—C1—S1	179.70 (9)	C5—C6—C11—C10	−179.51 (12)
N4—N3—C1—S1	−1.12 (18)	N1—C3—C12—C17	29.90 (17)
N1—N2—C2—N3	0.73 (13)	C4—C3—C12—C17	−93.44 (14)
N1—N2—C2—C18	178.95 (11)	N1—C3—C12—C13	−153.36 (12)
C1—N3—C2—N2	0.16 (14)	C4—C3—C12—C13	83.30 (15)
N4—N3—C2—N2	−179.04 (11)	C17—C12—C13—C14	1.6 (2)
C1—N3—C2—C18	−178.13 (11)	C3—C12—C13—C14	−175.28 (12)
N4—N3—C2—C18	2.68 (18)	C17—C12—C13—Cl1	−177.57 (10)
C1—N1—C3—C12	102.95 (14)	C3—C12—C13—Cl1	5.53 (17)
N2—N1—C3—C12	−86.96 (13)	C12—C13—C14—C15	−0.1 (2)
C1—N1—C3—C4	−133.35 (12)	Cl1—C13—C14—C15	179.07 (11)
N2—N1—C3—C4	36.74 (14)	C13—C14—C15—C16	−0.8 (2)
N1—C3—C4—C5	66.84 (13)	C14—C15—C16—C17	0.2 (2)
C12—C3—C4—C5	−167.78 (10)	C15—C16—C17—C12	1.4 (2)
C3—C4—C5—O1	6.81 (17)	C13—C12—C17—C16	−2.24 (19)
C3—C4—C5—C6	−176.50 (10)	C3—C12—C17—C16	174.63 (12)
O1—C5—C6—C11	−162.34 (12)	N2—C2—C18—C19	15.46 (18)
C4—C5—C6—C11	20.98 (17)	N3—C2—C18—C19	−166.53 (12)
O1—C5—C6—C7	18.16 (18)		

### Hydrogen-bond geometry (Å, °)

**Table:**

Cg is the centroid of the C12–C17 ring.

**Table:**

*D*—H···*A*	*D*—H	H···*A*	*D*···*A*	*D*—H···*A*
N4—H4B···S1	0.892 (18)	2.763 (16)	3.2239 (16)	113.5 (12)
N4—H4A···S1^i^	0.914 (18)	2.608 (18)	3.4773 (16)	159.2 (14)
N4—H4A···O1^i^	0.914 (18)	2.602 (18)	2.9123 (16)	100.6 (14)
N4—H4B···O1^i^	0.892 (18)	2.466 (15)	2.9123 (16)	111.3 (12)
C10—H10···Cg^ii^	0.95	2.62	3.508 (2)	156

Symmetry codes: (i) −*x*+1/2, −*y*+1/2, −*z*+1; (ii) −*x*, *y*, −*z*+3/2.
